# Determination of testicular estrogen receptor alpha expression of male chickens (*Gallus domesticus*) with age

**DOI:** 10.14202/vetworld.2019.994-997

**Published:** 2019-07-08

**Authors:** W. K. Ramesha Nirmali, Lakshan Warnakula, Ruwini Cooray, Nimanie Sachithra Hapuarachchi, Manjula P. S. Magamage

**Affiliations:** 1 Laboratory of Reproductive Biology and Animal Biotechnology, Department of Livestock Production, Faculty of Agricultural Sciences, Sabaragamuwa University of Sri Lanka, Belihuloya, Sri Lanka; 2 Section of Genetics, Institute for Research and Development, Colombo, Sri Lanka

**Keywords:** age, chicken, estrogen receptor alpha, gene expression, testicular

## Abstract

**Background and Aim:**

Estrogen activity, a central component of reproductive growth, is regulated by the receptor proteins, estrogen receptor alpha (ERα), and ER beta (ERβ) in chickens as in many other species. ERα expresses predominantly in gonads. Although the expression of ERα in embryonic gonads has been studied in detail, the expression of ERα in post-hatching male gonads has not been studied adequately. Therefore, the current research was conducted to determine the post-hatching changes in the expression of ERα in the left gonads of male chickens with age.

**Materials and Methods:**

Shaver Brown male chickens were raised and cared for according to the management guide and sacrificed at the intervals of 1, 4, and 8 weeks of age. The total RNA was extracted from the left gonads using the Trizol method and reverse transcribed using a pair of gene-specific primers. Following polymerase chain reaction amplification, the expression of ERα was quantified relative to the expression of the reference gene GAPDH.

**Results:**

The results showed that ERα expression significantly increases with age at p=0.0032. However, the increment of ERα expression from week 1 to week 4 was 2.04-fold and from week 4 to week 8 was 1.39-fold, with the later age reflecting a diminishing pattern in the increment.

**Conclusion:**

These results differentiate the post-hatching ERα expression of the left gonads of male chickens increase with age but with a diminishing gradient that may support their reproductive functions in later stages of life.

## Introduction

Estrogen is the primary female sex hormone which is responsible for the development and regulation of the female reproductive system and secondary sex characteristics [1]. Cellular estrogen activity is regulated by receptor proteins called estrogen receptors (ERs) which has two subtypes in vertebrates, ER alpha (ERα) and ER beta (ERβ) [2,3]. Research over the past two decades shows estrogen also plays a key role in the development and regulation of the male reproductive system [4]. Aromatase is the crucial enzyme responsible for the synthesis of estrogens by aromatization of the androgens [5]. Synthesis of estrogen and the relationship of estrogen and male reproductive performances have been previously studied in many species [6,7]. In the testes of adult roosters, estrogen is secreted by Leydig cells and immature germ cells where the aromatase gene is expressed [6]. In developing embryonic gonads, the expression of ERα has been observed differently in males and females (higher in females) while ERβ expressed indifferently in the two sexes demonstrating that α-type is more sex specific in chicken gonads [2]. The embryonic studies show that the early embryonic gonads (bipotential gonads) display ERα expression in the left but not in the right gonads of both sexes before gonadal differentiation [8]. Following gonadal differentiation (embryonic day 5), the expression of ERα gradually diminishes in males while expresses at higher concentrations in females [2,9-11]. These findings facilitate the previous argument that the ERα receptor is more sex specific in chickens. Further studies, therefore, are necessary to elucidate the actual role of ERα in the development of the reproductive system of male chickens.

Although the gonads show a diminishing trend of ERα during embryonic stage, the research involved in ERα expression of adult testes shows that the testicular and epididymal regions contain varying amounts of ERα reflecting its importance in the reproductive development of chickens during later stages of life [12]. This contradiction creates a research gap with the variation in the ERα expression in the testicular region of chickens with age. Understanding this variation will provide important insights about the activity and the requirement of estrogen within the male gonads toward reproductive development with age. According to literature, when birds are exposed to xenoestrogens, it is said to cause feminizing effects in the genetic male birds causing reproductive failures [13].

Therefore, to maximize male chicken reproductive potential, it is important to determine the natural level of ERα expression in the male gonads to identify the abnormal estrogen activity which could be detected by elevated ERα expression due to exogenous estrogen exposure in birds. This study was aimed to investigate the variation of the ERα expression in the left gonads of male chickens with age.

## Materials and Methods

### Ethical approval

All animal experiments were conducted under the guidance and approval of the Institutional Animal Care and Use Committee recommendations of Sabaragamuwa University of Sri Lanka.

### Care of chicks

All chicks were raised according to the guidelines given in the Shaver Brown Management Guide [14].

### Sacrificing chickens and sample collection

Three chicks were sacrificed at the age of 1 week, 4 weeks, and 8 weeks by inserting an air bubble through the brachial vein using a 1 mL syringe attached to a sterile 29 gauge needle. Following sacrifice, the carcasses were dissected and the testes were removed. The left testes were immediately placed in a small volume of Phosphate-buffered saline and transported on ice to the laboratory for further analysis.

### RNA extraction and quantification

The total RNA from the frozen left testes was extracted by the FavorPrep™ Tri-RNA Reagent (FAVORGEN Biotech Corp., Taipei, Taiwan) using the manufacturer’s protocol with slight modifications [15]. Extracted RNA was quantified at 260 nm wavelength using a Nanodrop 2000 spectrophotometer (Thermo Scientific, USA). Purity of the samples was determined by 260/280 and 260/230 ratios.

### Reverse transcription (RT) and cDNA synthesis

Extracted RNA of ERα was reverse transcribed using a gene-specific primer set described by Sakimura *et al*. [2] (Table-1). The reaction was performed using the FireScript® RT Kit (Solis BioDyne, Estonia) according to the FireScript Kit datasheet protocol [16]. The resulting cDNA was stored at −20°C until future RT polymerase chain reaction (PCR) analysis was conducted.

**Table 1:** Primer details of ERα expression analysis [2].

Fragment name	Amplicon size(bp)	Primer sequence(5’ to 3’)	Temperature(°C)
ERa forward	300	GTGCCTTAAGTCCATCATCCT	59.4
ER reverse		GCGTCCAGCATCTCCAGTAAG	63.3
GAPDH forward	348	GTGGAGAGATGACAGAGGTG	60.5
GAPDH reverse		AACAAGCTTGACGAAATGGT	54.3

ERa=Estrogen receptor alpha

### PCR amplification of ERα

About 1 µL of the RT product (cDNA) was used to amplify the ERα sequence using the forward and reverse primers previously described by Sakimura *et al*. [2]. PCR was performed with initial denaturation at 94°C for 2 min, followed by 40 cycles of denaturation at 97°C for 10 s, annealing at 55°C for 30 s, and extension at 72°C for 1 min and final extension for 2 min at 72°C.

### PCR amplification of GAPDH

GAPDH was used as the reference gene to quantify the relative expression of ERα. About 1 µL of the RT product from each sample was amplified. Initial denaturation was conducted at 94°C for 2 min, followed by 40 cycles of denaturation at 97°C for 10 s, annealing at 50°C for 30 s, and extension at 72°C for 1 min and a final extension for 2 min at 72°C.

### Agarose gel electrophoresis

PCR amplicons were electrophoresed in 1.5% agarose gel, which was prestained with 30,000 times diluted diamond dye in 1× TBE buffer. Electrophoresis conditions were 60 V for 1.5 h, and the DNA was visualized directly on a blue light transilluminator.

### Relative quantification of ERα expression and statistical analysis

ERα expression was semi-quantified using Image J (National Institutes of Health and the Laboratory for Optical and Computational Instrumentation, USA), an image processing software, and the mean intensity of the bands was used for the expression analysis. ERα expression as a percentage of the GAPDH expression was calculated in each sample using the mean intensity values. Relative expressions were statistically analyzed using a one-way ANOVA procedure of Statistical Analysis Software (SAS version 9.0) (SAS Institute, USA) to test the effect of age on the ERα expression in the left gonads of the male chickens.

## Results and Discussion

**Figure-1:**
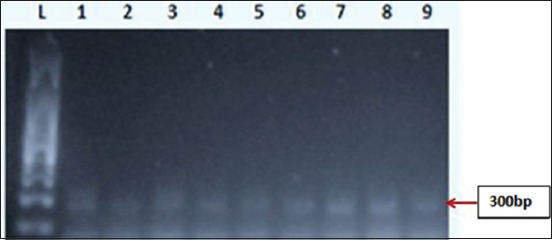
Electrogram for estrogen receptor alpha (ERα) expression in chicken gonads. L: 100 bp ladder, 1-3: ERα expression of week 1 chickens, 4-6: ERα expression of week 4 chickens, 7-9: ERα expression of week 8 chickens.

**Figure-2:**
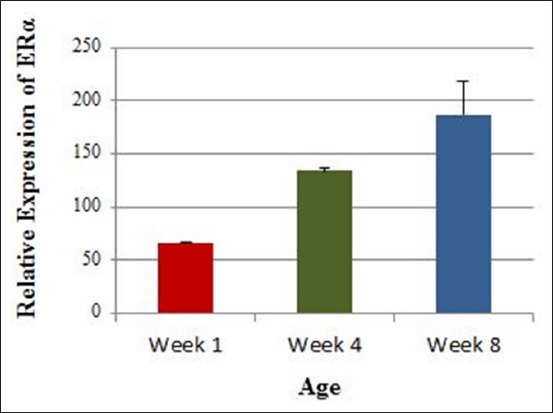
Variation of estrogen receptor expression with age.

The relative expression of ERα showed a significant increase with age (Figures-1 and 2). Statistically it was significant with p-value of 0.0032. The least square mean comparison test specified that there is a significant difference between each age interval (Table-2). These results provide evidence of the rise in ERα expression in the left gonads of male chickens with age.

**Table-2:** p-values for least squares mean comparison for male chicken.

p-values	Week 1	Week 4	Week 8
Week 1		0.0133	0.0011
Week 4	0.0133		0.0220
Week 8	0.0011	0.0220	

This finding contradicts the embryonic ERα expression results, which showed a diminishing expression of the ERα in male left gonads following gonadal differentiation. Therefore, this embryonic expression reduction can be interpreted as a sex-specific mechanism to support the differentiation of the bipotential gonads based on their genetic sex. Nevertheless, this finding is in compliance with the previous finding by the Gonzalen-Moran *et al*. [17], which displayed the highest ERα expression in testicles of mature chickens than in immature chickens showing an increment of ERα expression with age.

The post-hatching increase of the ERα expression in the gonads can be justified to support the increased activity of the estrogen in gonads for the reproductive development in male chicken with age, as per the latest finding of the role of estrogen in the male reproductive system observed in many species [4,18,19]. The studies involved in male infertility and ER knocked down mice models revealed that estrogen activity which is crucial in spermatogenesis and seminal fluid secretion is mediated by the ERα and hence holds utmost importance in the mammalian male testes [19,20]. A similar role of ERα expression could be suggested for avian model too with the results of the current study.

However, a study on domestic goose provided contrasting evidence to this which showed an inverse proportionate of ERα expression in testes with the plasma estrogen concentration during annual reproductive cycle [7]. Conversely, the sex reversal trials of chicken model suggested an induced expression of ERα in the embryonic male left gonads with an *in ovo* estradiol treatment [2,8]. This contradiction creates a gap for further research to check the variation of expression and the role of ERα in the testes of male birds with the age.

The experiment duration of the current study was 8 weeks and the sexual maturity of male chickens usually attains at 16 weeks of age [21]. Therefore, the results of the current study give an idea about the variation of the ERα expression during the presexual maturity period of chickens. The results also revealed that the post-hatching increase of ERα expression shows a diminishing pattern, by increasing from week 1 to week 4 in 2.04-fold and from week 4 to week 8 in 1.39-fold. This result gives speculation that the testicular ERα expression in chickens increases with age and comes to a peak at a certain age where the optimum reproductive development is facilitated and then declines or remains constant thereafter.

However, the previous research has evidence that the highest number of Sertoli cells and Leydig cells was found in immature chicken testicles, and the number drastically drops with the age [17]. Therefore, with the previous evidence of ERα expression confining to Sertoli cells and Leydig cells, the diminishing pattern of the increment of ERα expression can be explained as a result of the diminishing of these cells in the growing testicles. Although with a diminishing gradient, the increase of ERα expression with age can be suggested to support the other mechanisms behind the reproductive development such as production of seminal fluid and regulation of spermatogenesis. Nevertheless, it was also found that ERα expression was higher at the middle age than the aged chickens while there can be low amount of germ cells in aged chickens compared to middle-aged [17]. Combining the evidence from all studies, it can be suggested that the post-hatching ERα expression increases with a diminishing gradient up to a peak at a certain age and then it again declines with age corresponding to the reduction of the reproductive performance of chickens. However, further studies are essential to determine the role of ERα in the male reproductive development of the chicken model.

## Conclusion

Considering the results obtained from this research, it can be suggested that the ERα expression in the male left testes increases with age with a diminishing pattern of the increment during the presexual maturity period of chickens.

## Authors’ Contributions

WKRN contributed to conceptualization, investigation, data curation, and original manuscript draft. LW helped with the methodology, investigation, funding acquisition, data curation, and editing. RC and NSH helped with the methodology, investigation, analysis, and validation. MPSM contributed to conceptualization, funding acquisition, supervision, visualizations, editing, and review. All authors corrected the manuscript and read and read and approved the final manuscript.
